# The relationship between high density lipoprotein subclass profile and plasma lipids concentrations

**DOI:** 10.1186/1476-511X-9-118

**Published:** 2010-10-17

**Authors:** Li Tian, Mingde Fu

**Affiliations:** 1 Laboratory of Endocrinology and Metabolism, West China Hospital, Sichuan University, Chengdu 610041, Sichuan, People's Republic of China; 2 State Key Laboratory of Biotherapy, Sichuan University, Chengdu 610041, Sichuan, People's Republic of China

## Abstract

HDL particles posses multiple antiatherogenic activities and the identification and differentiation of individual HDL subclasses may be useful in documentation and understanding of metabolic changes of different HDL subclasses. The major plasma lipids exist and are transported in the form of lipoprotein complexes. Hence, alterations in plasma lipids levels can interfere with the composition, content, and distribution of plasma lipoprotein subclasses that affect atherosclerosis risk. The research review major discussed the relationship between plasma lipids levels and HDL subclasses distribution. The general shift toward smaller size of HDL particle size in HTG, HCL and MHL subjects, and the changes were more prominent with the elevation of TG and TC levels which imply that HDL maturation might be abnormal and RCT pathway might be weaken, and these changes were more seriously in MHL subjects. Plasma contents of small sized HDL particles significantly higher, whereas those of large sized HDL particles were significantly lower with elevation of TG/HDL-C and TC/HDL-C ratios. Increased in the TC/HDL-C ratio alone did not influence the distributions of HDL subclasses significantly when the TG/HDL-C ratio was low (TG/HDL-C ≤ 2.5). Hence, the TG/HDL-C ratio might be more sensitive to reflect the alteration of HDL subclass distribution than the TC/HDL-C ratio. In LDL-C/HDL-C ≤ 2.3 group, the pattern of distribution in HDL subclass was in agreement with the normolipidemic subjects. Moreover, considering the relative ease of measuring TC/HDL-C, TG/HDL-C and LDL-C/HDL-C ratios, as opposed to measuring HDL subclasses, these 3 ratios together may be a good indicator of HDL subclass distribution. The protective effect of increased apoA-I levels against the reduction of HDL_2b _caused by elevated TG concentration. On one hand, plasma HDL-C and apoA-I appear to play a coordinated role in the assembly of HDL particles and the determination of their contents among the total subjects. On the other hand, the apoA-I level might be a more powerful factor than HDL-C to influence the distribution of HDL subclasses in hyperlipidemic subjects. At the same time, from point of HDL subclasses distribution, the plasma lipids, apos concentrations and apos ratios should be considered while assessing the CHD risk. Abnormality of HDL subclasses distribution may result in accelerated atherosclerosis, therapeutic normalization of attenuated antiatherogenic HDL function in terms of both particle number and distribution of HDL particles is the target of innovative pharmacological approaches to large-sized HDL particles rising, including enhanced apoA-I levels.

## Introduction

It is well known that HDL does not represent a sum of identical particles but is rather comprised of discrete subclasses that differ related to charge, density, size, composition, shape and physiological functions [1]. Using two-dimensional gel electrophoresis coupled with immunoblotting, HDL can be divided into large, cholesterol-rich (HDL_2a _and HDL_2b_), small-sized (HDL_3c_, HDL_3b_, HDL_3a_, and preβ_1_-HDL) and preβ_2_-HDL [2,3]. Epidemiological studies have shown that individual HDL subclasses are not equally atheroprotective [4], a decrease content of the large-sized HDL_2b _particles and an increase content of the small-sized preβ_1_-HDL particles were highly and significantly associated with the risk of coronary heart disease(CHD)[5,6].

Plasma lipoproteins have the general structure. The hydrophobic cholesterol ester(CE) and triglyceride(TG) occupy a core that is surrounded by a hydrophilic monomolecular surface layer of apolipoproteins(apos), phospholipids(PL) and unesterified cholesterol[7]. So, the abnormalities of plasma lipids and apos transport in plasma will influence the composition and metabolism of lipoproteins.

We have systematic illustrated the characteristics of HDL subclasses distribution in different types of hyperlipidemic, obese subjects along with the effect of plasma lipids ratio on HDL subclasses distribution [3,8-15]. The characteristic of the transformation of HDL subclasses in these patients appeared to be different, whereas there was a general shift toward smaller sized HDL (preβ_1_-HDL increased while HDL_2a _and HDL_2b _decreased). Furthermore, the association between the relative concentrations of apos and the various HDL subclasses also investigated and reviewed [16-20]. The most significant association was observed between the contents of all HDL subclasses, especially large-sized HDL_2b _and apoA-I. ApoA-II played a dual function in the contents of HDL subclasses, and both small-sized HDL_3b _and HDL_3a _and large-sized HDL_2b _tended to increase with apoA-II concentration. An increase in the concentrations of apoC-II, C-III, and B-100 resulted in higher levels of small-sized HDL particles and lower levels of large-sized HDL particles. Higher concentrations of apoA-I could inhibit the reduction in the content of large-sized HDL_2b _effected by apoB-100, C-II, and C-III. The content of preβ_1_-HDL increases significantly and that of HDL_2b _declines progressively with an increased apoB-100/apoA-I or a decreased apoC-III/apoC-II ratio. In this research review mainly describes the effect of plasma lipids concentrations on HDL subclasses distribution profile.

## Characteristics of distribution of HDL subclasses for different types of hyperlipidemia

**Figure 1:**
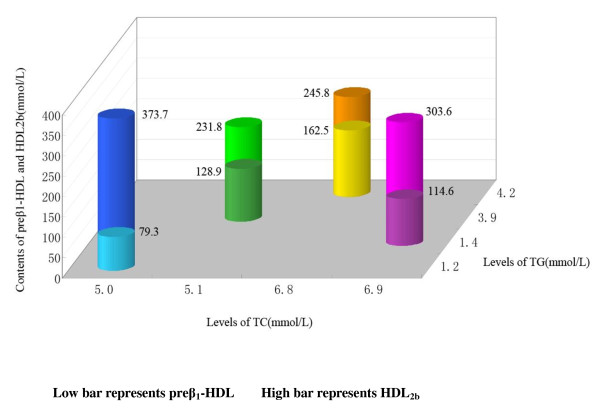
**Characteristics of major HDL subclasses (preβ_1_-HDL, and HDL_2b_) distribution in normolipidemia, endogenous hypertriglyceridemic, hypercholesterolmic along with mixed hyperlipidemic subjects**. This chart contains data from references [3,8,9], the mean of TG, and TC in normolipidemia, hypertriglyceridemia, hypercholesteromia, and mixed hyperlipidemia subjects, respectively, and their corresponds levels of preβ_1_-HDL and HDL_2b _were used to depict this chart. The mean TG and TC levels in normolipidemic were 1.2 and 5 mmol/L, in hypertriglyceridemia were 3.9 and 5.1 mmol/L, in hypercholesteromia were 1.4 and 6.9 mmol/L, and in mixed hyperlipidemia were 4.2 and 6.8 mmol/L, respectively. Compared with normolipidemia subjects, the contents of preβ_1_-HDL increased significantly, however, those of HDL_2b _decreased significant in hypertriglyceridemia, hypercholesteromia, and mixed hyperlipidemia subjects. Moreover, Preβ_1_-HDL increased most prominent in mixed hyperlipidemia subjects, and HDL_2b _decreased more apparent occurred in hypertriglyceridemia.

HDL metabolism is substantially altered in dyslipidemic states, including hypertriglyceridemia (HTG), hypercholesterolemia (HCL), and mixed dyslipidemia (MHL). The Figure 1 presented that the contents of major HDL subclasses (preβ_1_-HDL, and HDL_2b_) distribution in normolipidemia, endogenous HTG, HCL along with MHL subjects. Our previous investigations have found that although there were some minor differences of HDL subclasses distribution in different types hyperlipidemia subjects, the common tendency of increased small-sized and decreased large-sized HDL particles was observed [3,8,9]. Aida, et al. [21] test found that large subspecies (HDL_2b_, HDL_2a_) were lower, and small (HDL_3b_, HDL_3c_) were higher in hyperlipidemia group than in normolipidemia group.

### Endogenous hypertriglyceridemia and HDL subclasses distribution profile

Endogenous HTG is commonly encountered hyperlipidemia in clinical practice, which is characterized by elevation of TG and usually normal levels of TC. It is reported that more than half of Chinese patients with hyperlipidemia are endogenous HTG subjects. The possible reason is that diets high in carbohydrate are prevalent in China, which may result in disorder of TG metabolism (excessive production and/or deficient clearance) [22]. Epidemiologic studies provide increasing evidence that plasma TG level is an important independent risk indicator of CHD [23,24]. Miida, et al. [25] showed that increased preβ_1_-HDL contents at the expense of HDL_2b _in HTG. Meanwhile, it is also reported that a major subpopulation of HTG HDL had a mean diameter of 8.4 ± 0.1 nm (HDL_3_) [26]. Some studies also found that carbohydrate restriction significantly reduced TG involved in TG metabolism, reduced the levels of atherogenic lipoprotein particles, and increased the large HDL particle size [27].

After a series of screening criteria, 176 subjects with plasma TC < 6.21 mmol/L and TG ≥ 2.26 mmol/L were defined as HTG subjects, small-sized preβ_1_-HDL and HDL_3a _were significantly higher; however, large-sized HDL_2a _and HDL_2b _were significantly lower in HTG subjects versus normolipidemic subjects. In addition, males had significantly higher small-sized preβ_1_-HDL and HDL_3b_, but lower large-sized HDL_2b _than females in both normolipidemic and HTG subjects [8].

To study the influence of plasma different TG levels on HDL subclasses distribution, we divided the HTG subjects into 2 groups, that is, high (2.26-5.64 mmol/L), and very high ( ≥ 5.65 mmol/L) TG subjects; the normolipidemic subjects(TG < 2.26 mmol/L and TC < 6.21 mmol/L) classified as normal (<1.69 mmol/L) and borderline-high (1.69-2.25 mmol/L) TG subjects[8], according to the third Report of NCEP (ATP-III) guidelines[28], and observed that with the elevation of TG levels, the contents of small-sized preβ_1_-HDL and HDL_3a _increased successively, but those of large-sized HDL_2a _andHDL_2b _decreased successively. Compared with normal TG subjects, an increase (17%, 61%, and 124%) in preβ_1_-HDL and a decrease (7%, 37%, and 52%) in HDL_2b _were found in borderline-high, high and very high TG subjects, respectively [8].

Mechanisms leading to reduced large sized HDL particles and increased small sized HDL particles in HTG states are as follows: there is an increased mass transfer of TG from TG-rich lipoproteins(TRL) to HDL particles through the action of cholesteryl ester transfer protein (CETP) [29], a process leading to TG enrichment of HDL. TG-enriched HDL has been shown to be more prone to lipolysis by hepatic lipase (HL), giving rise to the formation of small, lipolytically modified HDL particles [30]. Moreover, some evidences have established that with the increase of plasma TG concentration, Lecithin-cholesterol acyltransferase (LCAT) and lipoprotein lipase (LPL) activities were impaired [31,14]. LCAT may catalyze unesterified cholesterol to cholesterol ester and promotes the conversion of preβ_1_-HDL and HDL_3 _to HDL_2_. LPL plays an important role in hydrolyzing TG transported in chylomicrons (CM) and very low density lipoprotein(VLDL) particles. When catabolized by LPL, CM and VLDL release TG, TC, phospholipids, apoA-I, and apoC. Subsequent binding of these products to HDL_3 _results in formation of HDL_2 _particles. Therefore, impaired LCAT and LPL activities reduce esterification of HDL-free TC, which resulted in abnormal HDL maturation and consequently higher preβ_1_-HDL and HDL_3a_, as well as lower HDL_2a _and HDL_2b_. All these observations demonstrated that plasma concentrations of TG have significantly important effects on the distributions of HDL subclasses and HDL maturation might be abnormal and RCT might be weakened in HTG subjects.

### Hypercholesterolemia, combined hyperlipidemia HDL subclasses distribution profile

HCL and MHL are common forms of atherogenic dyslipoproteinemia. HCL is characterized by elevation of TC and usually normal levels of TG while MHL is characterized by concomitant increase of plasma levels of TC and TG. Patients with HCL and with MHL have different lipid phenotypes, and HDL metabolism is substantially altered in these dyslipidemic states, however, are associated with accelerated atherosclerosis (As) [32].

By studying the characteristics of HDL subclasses distribution in HCL and MHL subjects, we found that both in HCL and MHL subjects, all small HDL particles (pre-β_1_-HDL, HDL_3c_, HDL_3b _and HDL_3a_) were significantly elevated whereas large HDL particles (HDL_2a _and HDL_2b_) were significantly reduced. Compared with normolipidemic subjects, an increase (44.3%, and 104%) in preβ_1_-HDL and a decrease (24.6%, and 53.9%) in HDL_2b _were found in HCL and MHL subjects, respectively. The findings suggests that the trend toward smaller of HDL size for MHL subjects was more obvious than HCL subjects [30]. The HDL subclasses metabolism was also reported by Saidi, et al. [33] the study included 11 MHL and 11 HCL patients. In MHL compared with HCL patients, decreased HDL_2 _levels were related to both HDL_2b _and HDL_2a _subpopulations (-57% and -49%, respectively, *P *< .01 for both). Moreover, in the study of Hogue for example, the integrated HDL size was significantly smaller in the familial HCL group compared to controls. In each groups, men had smaller HDL particles than women observed [34].

Numerous investigations have confirmed that elevation of TC level results in increased CHD risk [28,35,36]. Most studies clearly showed that in HCL and MHL subjects, CETP activity was high while LCAT activity was low, which was associated with the increased plasma TC level in these persons [37-39].

Above-mentioned, changes in these enzyme activities resulted in raising preβ_1_-HDL and HDL_3a_, as well as decreasing HDL_2a _and HDL_2b_. Correlation analysis revealed [9] that after controlling for sex, age, weight and BMI, plasma TG, TC and LDL-C levels were positively and significantly correlated with small-sized preβ_1_-HDL while negatively and significantly correlated with large-sized HDL_2b _not only in HCL but in MHL subjects. In contrast, HDL-C levels showed positive correlation with HDL_2b _only within MHL subjects. The changes of HDL subclasses in MHL subjects also probably related to lower HDL-C level, which might lead to the decrease of LCAT activity and increase of CETP activity [40]. Consequently, reduction in HDL-C and increases in TG as well as TC resulted in the more marked increase of small sized HDL subclasses in MHL subjects.

**Figure 2:**
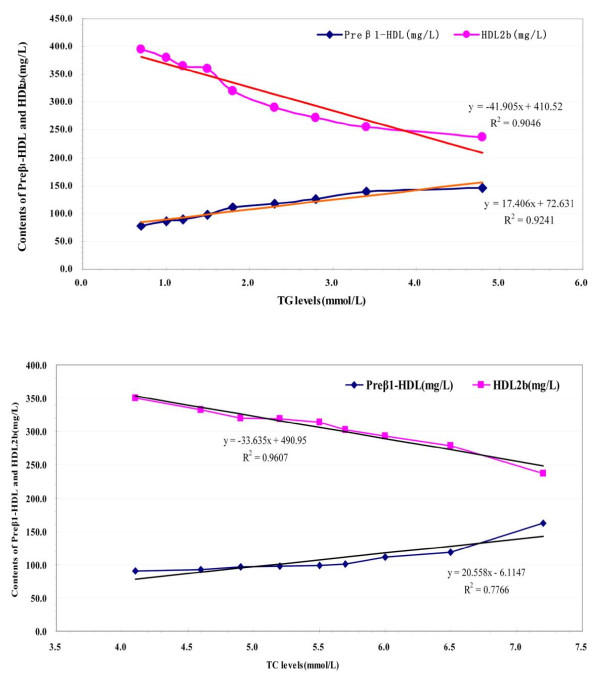
**The relationship between plasma TG, TC and HDL subclasses distribution**. For grouped analyses, individuals were classified according to approximately equal ninths of baseline TG and TC for the entire study population. Trends in mean values of major HDL subclasses (preβ_1_-HDL and HDL_2b_) across these ninths were assessed through simple linear regression, and using each stratum TG and TC median value as x axis, with every stratum TG and TC corresponding the median preβ_1_-HDL and HDL_2b _contents as y axis to plot. The results revealed that preβ_1_-HDL contents is elevated about 9 mg/L and HDL_2b _contents can be reduced 21 mg/L for 0.5 mmol/L increment in TG; unlikeness, the levels of TC were liner with HDL_2b _and HDL_2b _contents can be reduced 17 mg/L for 0.5 mmol/L increment in TC.

The TG and TC are two main types of lipids in plasma. Alteration of their concentrations predicts that the dynamic balance of plasma lipids metabolism was destroyed, which certainly will induce the changes in plasma lipoproteins along with their subclasses composition, contents and distribution. For grouped analyses, individuals were classified according to approximately equal ninths of baseline TG and TC for the entire study population (Unpublished data). Trends in mean values of major HDL subclasses (preβ_1_-HDL and HDL_2b_) across these ninths were assessed through simple linear regression, in this models with the contents of preβ_1_-HDL and HDL_2b _as the dependent variable and the levels of TG as independent variable (Figure 2), which exhibited that preβ_1_-HDL contents is elevated about 9 mg/L and HDL_2b _contents can be reduced 21 mg/L for 0.5 mmol/L increment in TG; unlikeness, the levels of TC were liner with HDL_2b _and HDL_2b _contents can be reduced 17 mg/L for 0.5 mmol/L increment in TC.

Above evidence showed that the general shift toward smaller size of HDL particle size in HTG, HCL and MHL subjects, and the changes were more prominent with the elevation of TG and TC levels. The changes mentioned above imply that HDL maturation might be abnormal and RCT pathway might be weaken, and which were more seriously in MHL subjects.

## Relationship between plasma lipids ratios and HDL subclasses distribution

**Figure 3:**
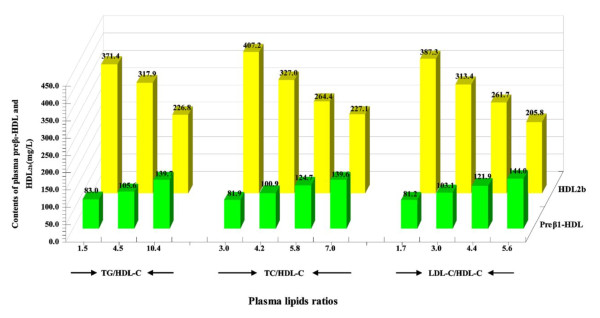
**Association of plasma lipids ratios and distribution of HDL subclasses**. This chart was constructed from data in references [8,11,12]. With the elevation of TG/HDL-C, TC/HDL-C, and LDL-C/HDL-C ratios, the contents of small-sized preβ_1_-HDL increased progressively, but those of large-sized HDL_2b _decreased progressively. Moreover, the characteristic of HDL subclass distribution for subjects with TG/HDL-C, TC/HDL-C, and LDL-C/HDL-C was 1.5, 3.0, and 1.7, respectively in accordance with that for the normolipidemic subject in our previous study. Especially, the subjects with LDL-C/HDL-C > 5.6 group had significant higher preβ_1_-HDL and significant lower HDL_2b_, which resulted in the ratio of HDL_2b_/preβ_1_-HDL from 4.8 in LDL-C/HDL-C < 1.7 group decreased to 1.4.

The Third Adult Treatment Panel guidelines of the US National Cholesterol Education Program (ATP-III) [28] recommend a full fasting lipoprotein profile, including TG, TC, HDL-C, and low-density lipoprotein cholesterol (LDL-C). Although the guidelines only provide for evaluation of individual lipid fractions, the application of ratios such as TC/HDL-C, TG/HDL-C, and LDL-C/HDL-C may offer a refined risk assessment by simultaneously considering both anti-atherogenic and atherogenic lipid parameters. The Figure 3 presented that the relationship between plasma lipids ratios and HDL subclasses distribution [11,12].

### Plasma TG/HDL-C and TC/HDL-C ratios and HDL subclasses distribution profile

Some literatures indicate that elevated fasting TG represent a useful marker for risk of CHD, particularly when HDL-C levels are considered [41]. The strong association of the ratio of TG/HDL-C with risk of CHD suggests a metabolic interaction between the TG- and cholesterol ester-rich lipoproteins in increasing risk of myocardial infarction (MI) [41]. Ratio of cholesterol ester-rich lipoprotein levels (TC/HDL-C) is well-established predictors of CHD [42], and a high ratio may be a good indicator of abnormal cholesterol metabolism and a 1-unit increase in the TC/HDL-C associated with 49% increases in risk of MI. Da Luz, et al. [43] study indicates that TC/HDL-C ratio is an easy, non-invasive means of predicting the presence and extent of coronary atherosclerosis, and the ratio TG/HDL-C, initially proposed by Gaziano, et al. [41] is an atherogenic index that has proven to be a highly significant independent predictor of MI even stronger than TC/HDL-C.

According to the ATP-III, 3 ranges of TC are defined: less than 200, 200-240, and 240 mg/dL or greater; similarly, 3 ranges of TG are defined: less than 150,150-200, and 200 mg/dL or greater. In addition, low HDL-C levels are designated as less than 40 mg/dL and high levels as 60 mg/dL or greater[28]. In recent years, it has been reported that risk for cardiac events is significantly higher when the TC/HDL-C ratio is greater than 5; Therefore, we make use of the TC/HDL-C ratio of 3.3 (200/60) and 6 (240/40) as the cutpoints and insert an additional TC/HDL-C group ranging from 3.3 to 5 [11]. The subjects were divided into TC/HDL-C ≤ 3.3, 3.3 < TC/HDL-C ≤ 5, 5 < TC/HDL-C ≤ 6, and TC/HDL-C > 6 groups. Likewise, TG/HDL-C ratios of 2.5 (150/60) and 5 (200/40) were used as the cutpoints. The subjects were divided into 3 groups: TG/HDL-C ≤ 2.5, 2.5 < TG/HDL-C ≤ 5 and TG/HDL-C > 5 group [11].

Analyzing the relationship between the ratios of TC/HDL-C, TG/HDL-C, and the alteration of HDL subclasses showed that, with the elevation of these ratios, HDL particles shifted toward smaller sizes [35]. Meanwhile, the characteristic of HDL subclass distribution for subjects with TC/HDL-C ≤ 3.3 or/and TG/HDL-C ≤ 2.5 was in accordance with that for the normolipidemic subject in our previous study [8,9,11,12]. Data obtained also suggest that, compared to the TC/HDL-C ≤ 3.3 along with TG/HDL-C ≤ 2.5 group, small-sized preβ_1_-HDL increased significantly, whereas large-sized HDL_2b _decreased significantly, which resulted in an amazing reduction of HDL_2b_/preβ_1_-HDL ratio (1.1 vs 4.7) and the percentage of large-sized HDL subclasses (31.8% vs 53.4%) in the TC/HDL-C > 6 along with TG/HDL-C > 5 group [11]. These findings revealed that subjects with the high ratios of TC/HDL-C and TG/HDL-C might have increased risk of CHD, because the RCT might be weakened and the potential anti-atherogenic functions of HDL might be impaired seriously among these subjects.

As to the influence of change in the TC/HDL-C ratio or the TG/HDL-C ratio on the apoA-I contents of preβ_1_-HDL and HDL_2b _which revealed that in the TG/HDL-C ≤ 2.5 groups [11], regardless of whether the TC/HDL-C ratios increased or not, both the preβ_1_-HDL and HDL_2b _were almost kept at constant levels. However, in each same TC/HDL-C ratio group, preβ_1_-HDL increased significantly, whereas HDL_2b _decreased significantly with the increase in the TG/HDL-C ratios. These observations implication that increase in the TC/HDL-C ratio alone did not influence the distributions of HDL subclasses significantly when the TG/HDL-C ratio was low (TG/HDL-C ≤ 2.5). Hence, the TG/HDL-C ratio might be more sensitive to reflect the alteration of HDL subclass distribution than the TC/HDL-C ratio.

### Plasma LDL-C/HDL-C ratio and HDL subclasses distribution profile

There is overwhelming evidence [44,45] that an elevated LDL-C concentration in plasma is atherogenic, whereas a high HDL-C level is cardioprotective [45-47]. A series of studies do suggest that use of the ratio of LDL-C to HDL-C is superior to use of HDL-C or LDL-C alone [48] and the ratio of LDL-C/HDL-C may provide better risk assessment by concurrently accounting for both atherogenic and protective lipid fractions. In the Physicians' Health Study, a 1-unit increase in the LDL/HDL ratio was associated with a 53% increase in risk of MI [49].

To assess the association of the LDL-C/HDL-C ratio with HDL subclass distribution, The subjects categorized into 4 subgroups ( ≤ 2.3, 2.3 to 3.9, 3.9 to 4.6, and >4.6) based on the Quebec Cardiovascular Study and calculated odds ratios for ischemic heart disease (IHD) [12]. With the elevation of LDL-C/HDL-C ratio, the contents of small-sized preβ_1_-HDL, HDL_3a_, and HDL_3b _increased progressively, but those of large-sized HDL_2a _and HDL_2b _decreased progressively. In LDL-C/HDL-C ≤ 2.3 group, the pattern of distribution in HDL subclass was in agreement with the normolipidemic subjects [8]. Compared with LDL-C/HDL-C ≤ 2.3 group, an increase (50%, and 77%) in preβ_1_-HDL and a decrease (48%, and 88%) in HDL_2b _were found in 3.9 < LDL-C/HDL-C ≤ 4.6, and LDL-C/HDL-C > 4.6 groups, respectively [12]. Where it indicated that increased IHD risk in 3.9 to 4.6 and >4.6 of the LDL-C/HDL-C ratio (odds ratio 3.4 and 3.7, respectively) compared with IHD odds ratio for ≤ 2.3 along with 2.3 to 3.9 of LDL-C/HDL-C subgroups was 1.0 and 1.9, separately [50].

The HDL subclass distribution remodeling might explained by increased LDL-C, TG, and decreased HDL-C levels. The large majority of studies have implicated that enhanced CETP and HL activities are correlated with low HDL-C and high LDL-C levels [30,31,38,39]. On the other hand, a significant increase in TG levels with a rise in LDL-C/HDL-C ratio also observed in this study. It has been reported that high levels of TG are associated with impaired LPL and LCAT activities [31,14]. Take these all together suggested that high LDL-C/HDL-C ratio was associated with low levels of large-sized HDL_2 _and generally with small-sized HDL particles.

An ideal ratio of LDL-C/HDL-C was 3.5 has been used as markers of coronary atherosclerosis [51]. In this context, the values of 3.5 were selected for LDL-C/HDL-C ratio and ATPIII guidelines 2.26 mmol/L for TG [28]as critical cutoff points to examine the joint effect of LDL-C/HDL-C ratio together with TG levels on change in HDL subclass distribution, which exhibit that the particles of HDL subclasses tend to small in LDL-C/HDL-C ≥ 3.5 group versus LDL-C/HDL-C < 3.5 group which indicated that abnormal metabolism of HDL subclasses in LDL-C/HDL-C ≥ 3.5 group. Castelli, et al. [51] observed that average LDL-C/HDL-C ratio for people without CHD is less than 3.4, while value of the ratio among patients with excessive rates of CHD is 3.5 or greater. In this regard, it is important to point out that applying the LDL-C/HDL-C ratio to estimate the risk of CHD should combine with effect of individual TG levels.

Considering the relative ease of measuring TC/HDL-C, TG/HDL-C and LDL-C/HDL-C ratios, as opposed to measuring HDL subclasses, these 3 ratios together may be a good indicator of HDL subclass distribution (and, thus, cardiovascular disease risk).

## The effect of plasma lipids combined with apolipoproteins on HDL subclasses distribution

We have made a review of the association between the relative concentrations of apos and the various HDL subclasses [16-20]. The findings demonstrated that different apos have distinct influence on the profile of HDL subclasses distribution. In this research review, we discussed the effect of plasma lipids coordinates with apos on the profile of HDL subclasses distribution.

### Plasma TG, apoA-I concentrations and HDL subclasses distribution profile

On one hand, the TG-mediated alteration of HDL subclasses distribution involves specific interaction with plasma enzyme activities, protein factor, and so on. Another important metabolic trigger for HDL mature metabolic disorder in the elevated TG concentrations condition could be related to the plasma apos levels alter. As a major protein component (about 70%) of HDL, apoA-I promote cholesterol efflux from cells, and through this mechanism may be important in maintaining cellular cholesterol homeostasis [52]. Meanwhile, apoA-I is not only activator of LCAT but also is a critical ligand of the HDL receptor SR-BI and the interaction of apoA-I and SR-BI may facilitate hepatic selective uptake of HDL-C in the RCT pathway hence play a vital role in the maturation process of HDL subclasses [53,54]. The apoA-I concentrations divided into tertiles and TG levels by ATP-III guidelines to investigate the influence of TG combined with apoA-I levels on phenotype of HDL subclasses distribution (Unpublished data).

The findings presented that regardless of elevated TG and/or apoA-I, preβ_1_-HDL contents were obviously increased; Likeness, in any TG levels, the contents of HDL_2b _significantly and gradually increased with elevated of apoA-I levels, which suggested that the protective effect of increased apoA-I levels against the reduction of HDL_2b _caused by elevated TG concentration.

### Plasma HDL-C, apoA-I concentrations and HDL subclasses distribution profile

The studies also reported what degree of the HDL particles distribution was affected depend on a relatively change between in HDL-C and apoA-I concentrations [16]. All HDL subclasses contents increased gradually and significantly, with elevated apoA-I along with HDL-C levels, and the percentage of small-sized HDL subclasses increased was low relative to that of the large-sized HDL subclasses generally. Plasma HDL-C and apoA-I appear to play a coordinated role in the assembly of HDL particles and the determination of their contents [16]. On the side, in each same apoA-I group, regardless of the HDL-C concentrations both the preβ_1_-HDL and HDL_2b _were almost kept at the same level. However, in each same HDL-C group, preβ_1_-HDL and HDL_2b _increased significantly with the increase of apoA-I in hyperlipidemic subjects. It indicated that apoA-I level might be a more powerful factor to influence the distribution in different HDL subclasses [13]. Recently, small, amphipathic helical apoA-I mimetic peptides composed of D-amino acids have shown similar antiatherogenic properties. Moreover, a specific apoA-I mimetic peptide, D4F, has shown improved HDL-mediated efflux and RCT from macrophages [55-57].

### Plasma TG concentration, apoB-100/apoA-I ratio and HDL subclasses distribution profile

The large prospective apolipoprotein-related Mortality Risk (AMORIS) study [58] suggests that apoB-100, apoA-I and the apoB-100/apoA-I ratio should be regarded as highly predictive in evaluating cardiac risk [58,59]. A series of studies have shown that an apoB-100/apoA-I ratio ≥ 0.9 was a fair predictor of presence of the metabolic syndrome (MetS) [60], and men with an apoB-100/apoA-I ratio >0.9 had also a faster growth of carotid artery intima-media thickness (IMT) than those below this value [61]. Using a 0.9 cut-point for the apoB-100/A-I ratio to dichotomize and analyze the alteration of HDL subclasses distribution (Unpublished data), and found the preβ_1_-HDL contents increased significantly and the HDL_2b _contents decreased significantly as the elevation of TG concentration despite the subjects with the apoB-100/apoA-I ratio < 0.9. At the same time, in comparison with the normal TG, the marked lower values of HDL_2b_/preβ_1_-HDL in both high and very high TG groups (5.3 vs 2.3, 1.7). Similarly, in the apoB-100/apoA-I ratio ≥ 0.9, the HDL subclasses distribution might be reversed for subjects with normal TG concentration (Unpublished data). Although the cholesterol balance determined as the apoB-100/apoA-I ratio has repeatedly been shown to be a better index for risk assessment of CHD, from the point of view of HDL subclasses distribution, all these findings revealed that when evaluation the CHD risk, relying only on the apoB-100/apoA-I values for subjects might be inadequate and the concentration of TG should be concerned.

In addition, to divide apoC-II into tertiles among the whole population and the study showed compared to the lowest tertile of apoC-II, the subjects with the highest tertile of apoC-II represent a typical HTG lipids profile [17,18]. The higher TG concentrations were associated with the levels of apoC-II and apoC-III increased, and an increasing in apoC-II more prominent than in apoC-III which conduces to the apoC-III/C-II ratio declined. At the same time, there was a consistent trend of increasing in contents of preβ_1_-HDL whereas a decreasing trend of in those of HDL_2b _with the reduction of apoC-III/C-II value. We also classified the apoB-100 into tertiles in the total subjects and shed light on the impact of changes in plasma apoB-100 levels on HDL subclasses distribution. The findings were the concentrations of TC and TG were significantly increased and HDL particles tend to smaller for subjects in the highest tertiles compared with those in the lowest tertiles of apoB-100[19]. So, from point of HDL subclasses distribution, the plasma lipids together with apos concentrations should be considered while assessing the CHD risk.

The antiatherogenic properties of HDL can, however be compromised in dyslipidemic states associated with elevated cardiovascular(CV) risk, therapeutic normalization of attenuated antiatherogenic HDL function in terms of both particle number and distribution of HDL particles is the target of innovative pharmacological approaches to large-sized HDL particles raising, including enhanced apoA-I levels. Abnormality of HDL subclasses distribution may result in accelerated As, such normalization of HDL metabolism can result from the plasma lipids and apos alteration.

## Conflict of interests

The authors declare that they have no competing interests.

## Authors' contributions

LT participated in the design of study and manuscript preparation along with editing. MF conceived of the study, and helped to review the manuscript. All authors read and approved the final manuscript.
